# Descriptions of two new species in the genus *Macrostylis* Sars, 1864 (Isopoda, Asellota, Macrostylidae) from the Weddell Sea (Southern Ocean), with a synonymisation of the genus *Desmostylis* Brandt, 1992 with *Macrostylis*

**DOI:** 10.3897/zookeys.57.310

**Published:** 2010-09-21

**Authors:** Torben Riehl, Angelika Brandt

**Affiliations:** Biozentrum Grindel & ogisches Museum, Martin-Luther-King-Platz 3, 20146 Hamburg

**Keywords:** Antarctic, deep sea, Macrostylis uniformis sp. n., Macrostylis antennamagna sp. n., ANDEEP, Janiroidea, taxonomy, macrobenthos

## Abstract

Descriptions of Macrostylis antennamagna **sp. n.** and Macrostylis uniformis **sp. n.** are presented with notes on intraspecific variability and sexual dimorphism. Macrostylis uniformis **sp. n.** showes differences to Macrostylis antennamagna **sp. n.** in the length of the antenna 2, the shape of the pleotelson and length of uropods.

The genus Desmostylis Brandt, 1992 (formerly including the two species Desmostylis obscurus Brandt, 1992 and Desmostylis gerdesi Brandt, 2002) is synonymised with the genus Macrostylis. Based on type material additional remarks and additions to the original descriptions are provided for both species. Results lead to following nomenclatorial changes: Macrostylis obscurus (Brandt, 1992), **comb. n.** and Macrostylis gerdesi (Brandt, 2002), **comb. n.** A setal nomenclature is proposed and the diagnosis for the family is revised.

## Introduction

During recent ANDEEP I–III cruises (ANtarctic benthic DEEP-sea biodiversity: colonization history and recent community patterns (Brandt et al. 2004a)), isopods have been found to be an important component of the Southern-Ocean deep sea (in terms of both richness and abundance). Most of the collected isopods belonged to the suborder Asellota Latreille, 1802 (Brandt et al. 2004b, Brandt et al. 2007a). Among these, the family Macrostylidae Hansen, 1916 showed a remarkable species richness (Brandt et al. 2004, 2007, Kaiser et al. 2007).

Macrostylidae have been suggested to be a specialized endobenthic component of deep-sea macrofauna (Harrison 1989, Hessler and Strömberg 1989). They have been regularly reported from deep-sea samples (e.g. Menzies 1962, Wolff 1962, Menzies and George 1972, Brandt 2002, Wilson 2008, Vey and Brix 2009).

This taxon consists of two genera: Macrostylis Sars, 1864 and Desmostylis Brandt, 1992. Species of Desmostylis have been reported from the Antarctic shelf (Desmostylis gerdesi Brandt, 2002; 238 m) and deep sea (Desmostylis obscurus Brandt, 1992; 4335 m). Species of Macrostylis have been reported from all major marine realms, from near-shore and deeper sublittoral habitats (e.g. Macrostylis spinifera Sars, 1864 from 27–1761 m) to hadal regions (e.g. Macrostylis mariana Mezhov, 1993; 10223–10730 m, e.g. deepest isopod record), and thus Macrostylidae has the widest depth range amongst all isopod families (Tab. 2).

To date, 78 valid macrostylid species are known worldwide of which ten occur in the Southern Ocean (Tab. 2). During the ANDEEP cruises at least 33 species of Macrostylidae have been collected, of which 23 where unlike any previously described species (Vey and Brix 2009). In the current paper, two of these previously unknown species, Macrostylis unifomis sp. n. and Macrostylis antennamagna sp. n. are described. Based on type material, additions to the original description of Desmostylis obscurus is presented and close examination of characters led to a rejection of the genus Desmostylis. It has been found synonymous with Macrostylis.

## Material and methods

Specimens used for species descriptions were collected at four stations in the northern and south-eastern Weddell Sea during the ANDEEP II–III expeditions with RV Polarstern. These cruises took part in Austral summer 2001/2002 and 2004/2005. An epibenthic sledge was used for sampling (Brenke 2005 and references therein). On board samples were immediately transferred into 96% pre-cooled ethanol and stored at -20° for at least 48 h. Samples were sorted into major groups on board. Sorting of isopods to family level has been continued in the laboratories of the Zoological Museum, Hamburg.

For habitus drawings and dissections of limbs, specimens were transferred into glycerine. Habitus were photographed in deionized water or glycerine. For identifications and pencil habitus sketches a Leica MZ12.5 with a camera lucida was used (max. 100 ×). Specimens were stained in high-concentration water solution of methylene green or stained glycerine (glycerol with methylene green). Limbs and habitus of small specimens were drawn using a Leica DM 2500 with camera lucida (max. 800 × with phase contrast). For digital photographs a Keyence VHX-500FD digital microscope with two lenses (VH Z20R & VH Z100R) was applied. Limbs were fixed on temporal or permanent slides. Temporal slides were made using stained glycerine. Permanent slides were made using Hydro-Matrix. Pencil drawings were scanned as grayscale PDF. Line drawings were made from pencil drawings and stack photos using Adobe Illustrator and WACOM Intuos digitizer boards following Coleman (2003, 2009).

Ratios were calculated from measurements made from the line drawings. Measurements were made following the method of Hessler (1970). They were taken using the distance measurement and cumulative distance measurement tools imbedded in Adobe Acrobat Professional. All appendages´ article-length ratios are given in proximal to distal order, excluding setae. Body lengths are given in anterior-posterior order excluding appendages. Ratios were rounded to first position after decimal point. Only one or two specimens were precisely measured for each description. Thus, a certain number provided here guarantees only to be within an unknown range of variation.

**Figure 1.**
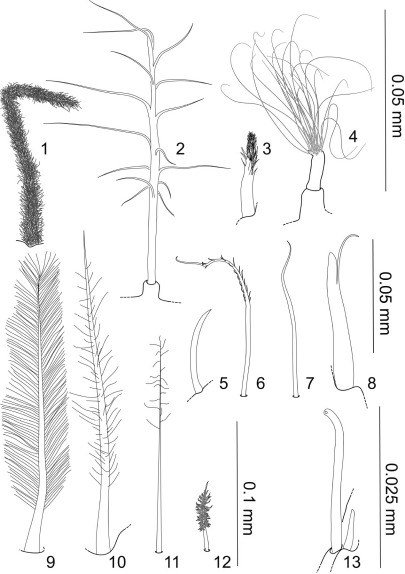
**1–13** Specialized setae, all articulating infracuticularly, except when mentioned otherwise; **1** Sensilla, pappose seta, shaft densely covered with fringe-like appearing setules; on pereopod 1–3 dactyli **2** broom seta, pedestal pappose seta **3** sensilla, distally pappose seta, densely covered with fringe-like appearing setules, showing a gradual transition from denticles to setules; on distal margins of maxilliped palp and on pereopods **4** tuft seta, a pedestal pappose seta, setule bases closely together, continuous transition to shaft cuticle; only known from distal articulation margin of 5. article in antenna 2 **5**, **7** simple setae, short **5** on general cuticle and pereopodal basis **6–8**, **12** different setal types, on lateral margin of pleopod cavity and operculum **6** plumose seta with few short setules on distal half **8** spine-like unequally bifid seta **12** small pappose seta, with short setules **9** “feather-like” plumose seta like at pleopod 3 apex **10–11** pappose setae, on operculum distal apex and pleopod 2 distal apex **13** simple tubular seta with big apical pore and associated small simple seta, on antenna 1 articles and antenna 2 flagellar articles.

**Figure 2.**
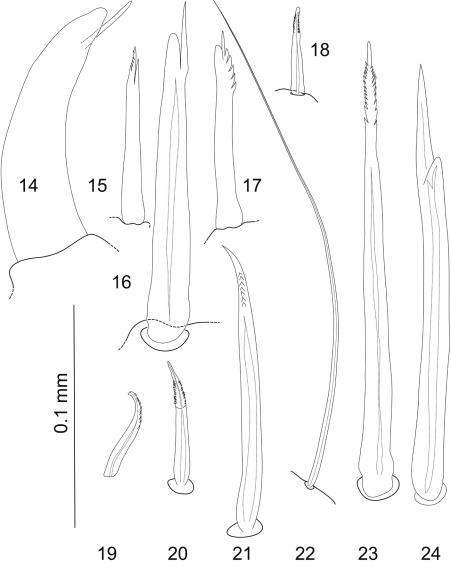
**14–24** Most common pereopodal setae, occurring e.g. on dorsal and ventral margins of all pereopodal articles; **14–16** unequally bifid setae **15**, **17**, **18–21**, **23** serrate setae **22** simple seta, very long and slender **24** bifurcate seta. Lumen indicated by dotted lines where visible in light microscopy. Scale bar = 0.1 mm.

Terminology is based on Hessler (1970), Wilson (1989) and Larsen (2003) with several additions and modifications. Setal nomenclature follows Hessler (1970) and is updated after Larsen (2003) and Garm (2004). See also Figs 1–2. Type material examined is listed in Table 1.

**Table 1.** Type material examined for comparison. Macrostylis longiremis (Meinert, 1890) is labelled as Vana longiremis Meinert, 1890. Macrostylis obscurus (Brandt, 1992) and Macrostylis gerdesi (Brandt, 2002) are labelled as the genus Desmostylis.

Scientific Name	Museum no	Type status
Macrostylis abyssalis Brandt, 2004	ZMH K-40284	Holotype
Macrostylis abyssalis Brandt, 2004	ZMH K-40285	Paratype
Macrostylis abyssicola Hansen, 1916	ZMUC CRU-5037	Syntypes
Macrostylis abyssicola Hansen, 1916	ZMUC CRU-5038	Syntypes
Macrostylis abyssicola Hansen, 1916	ZMUC CRU-5046	Syntypes
Macrostylis angolensis Brandt, 2004	ZMH K-40280	Holotype
Macrostylis angolensis Brandt, 2004	ZMH K-40281	Paratype
Macrostylis bifurcatus Menzies, 1962	AMNH 12126	Holotype
Macrostylis bifurcatus Menzies, 1962	AMNH 12268	Paratype
Macrostylis bipunctatus Menzies, 1962	AMNH 12056	Holotype
Macrostylis bipunctatus Menzies, 1962	AMNH 12057	Allotype
Macrostylis bipunctatus Menzies, 1962	AMNH 12262	Paratype
Macrostylis bipunctatus Menzies, 1962	AMNH 12277	Paratype
Macrostylis bipunctatus Menzies, 1962	AMNH 12265	Paratype
Macrostylis caribbicus Menzies, 1962	AMNH 12072	Holotype
Macrostylis cerritus Vey & Brix, 2009	ZMH K-41431	Holotype
Macrostylis cerritus Vey & Brix, 2009	ZMH K-41432	Paratype
Macrostylis cerritus Vey & Brix, 2009	ZMH K-41433	Paratype
Macrostylis cerritus Vey & Brix, 2009	ZMH K-41434	Paratype
Macrostylis dellacrocei Aydogan, Wägele & Park 2001	ZMB 27338	Holo- and Paratypes
Macrostylis elongata Hansen, 1916	ZMUC CRU6348	Holotype
Macrostylis galatheae Wolff, 1956	ZMUC CRU6509	Syntypes
Macrostylis gerdesi (Brandt, 2002) comb. n.	ZMH K-39915	Holotype
Macrostylis gerdesi (Brandt, 2002) comb. n.	ZMH K-39916	Paratype
Macrostylis hadalis Wolff, 1956	ZMUC CRU-6647	Holotype
Macrostylis hadalis Wolff, 1956	ZMUC CRU-6648	Allotype
Macroistylis hirsuticaudis Menzies, 1962	AMNH 12122	Holotype
Macroistylis hirsuticaudis Menzies, 1962	AMNH 12122A	Allotype
Macroistylis hirsuticaudis Menzies, 1962	AMNH 12123	Paratypes
Macrostylis longifera George & Menzies, 1972	USNM 121743	Non-type
Macrostylis longifera George & Menzies, 1972	USNM 121746	Allotype
Macrostylis longifera George & Menzies, 1972	USNM 121745	Holotype
Macrostylis longifera George & Menzies, 1972	USNM 121747	Paratype
Macrostylis longipedis Brandt, 2004	ZMH K-40278	Holotype
Macrostylis longipedis Brandt, 2004	ZMH K-40279	Paratype
Macrostylis longipes Hansen, 1916	ZMUC CRU-7126	Holotype
Macrostylis longispinis Brandt, 2004	ZMH K-40286	Holotype
Macrostylis longiremis (Meinert, 1890)	ZMUC CRU-7131	Syntype
Macrostylis longiremis (Meinert, 1890)	ZMUC CRU-9596	Syntype
Macrostylis magnifica Wolff, 1962	ZMUC CRU-7189	Holotype
Macrostylis meteorae Brandt, 2004	ZMH K-40282	Holotype
Macrostylis meteorae Brandt, 2004	ZMH K-40283	Paratype
Macrostylis minutus Menzies, 1962	AMNH 12059	Holotype
Macrostylis minutus Menzies, 1962	AMNH 12060	Paratype
Macrostylis minutus Menzies, 1962	AMNH 12203	Paratypes
Macrostylis minutus Menzies, 1962	AMNH 12202	Paratypes
Macrostylis obscurus Brandt, 1992 comb. n.	BM (NH) 1990:39:1	Holotype
Macrostylis robusta Brandt, 2004	ZMH K-40277	Paratype
Macrostylis robusta Brandt, 2004	ZMH K-40695	Paratype
Macrostylis robusta Brandt, 2004	ZMH K-40696	Paratype
Macrostylis robusta Brandt, 2004	ZMH K-40697	Paratype
Macrostylis sarsi Brandt, 1992	BM (NH) 1990:40:1	Holotype
Macrostylis setifer Menzies, 1962	AMNH 12058	Holotype
Macrostylis subinermis Hansen, 1916	ZMUC CRU-8301	Syntype
Macrostylis subinermis Hansen, 1916	ZMUC CRU-8302	Syntype
Macrostylis subinermis Hansen, 1916	ZMUC CRU-8303	Syntypes
Macrostylis subinermis Hansen, 1916	ZMUC CRU-8304	Syntypes
Macrostylis subinermis Hansen, 1916	ZMUC CRU-8305	Syntypes
Macrostylis subinermis Hansen, 1916	ZMUC CRU-8306	Syntype
Macrostylis trucatex Menzies, 1962	AMNH 12065	Holotype
Macrostylis vemae Menzies, 1962	AMNH 12074	Holotype
Macrostylis vemae Menzies, 1962	AMNH 12075	Allotype
Macrostylis vemae Menzies, 1962	AMNH 12076	Paratypes
Macrostylis vemae Menzies, 1962	AMNH 12204	Paratypes

### Abbreviations.

AMNHAmerican Museum of Natural History, New York City; NHMNatural History Museum, London, UK; NMNHNational Museum of Natural History, Washington, D.C.; ZMBMuseum für Naturkunde, Berlin, Germany; ZMHZoological Museum Hamburg, University of Hamburg, Germany; ZMUCZoologisk Museum, University of Copenhagen, Denmark.

## Taxonomy.

Asellota Latreille, 1802

Macrostylidae Hansen, 1916

### Desmosomidae

Sars, 1899

Macrostylini Hansen 1916 p. 74; Wolff 1956 p. 99
Macrostylinae Birstein, 1973
Macrostylidae Gurjanova 1933, p. 411; Menzies 1962a, p. 28; 1962b, p. 127; Wolff 1962, Menzies and George 1972, p. 79–81; Wägele 1989, Brandt 1992; 2002; 2004; Birstein 1970, Kussakin 1999, p. 336; Mezhov 1988, p. 983–994; 1992, p. 69

#### Composition.

Macrostylis Sars, 1864

#### Type genus.

Macrostylis Sars, 1864

#### Diagnosis.

Cephalothorax free, about as broad as long. Body elongated. Eyes absent. Antenna 1 small, number of articles variable (1–9). Antenna 2 short or only moderately long, with articles 1–3 together about as long as articles 4 and 5 respectively, squama absent. Mandible with pars molaris reduced, subacute triangular and setiferous on apex; palp absent; lacinia mobilis ambilateral. Maxilliped with long and narrow basis; subtriangular epipod subequal in length to basis; palp articles 1–3 expanded and articles 4–5 minute.

Pereonites 1–3 constituting separate subquadrangular section with tightly articulated segments but tergite borders visible: fossosome. Pereonites 4–7 articulated moveably, constricted anteriorly. Pereopods 1–4 fossorial; pereopods 1–3 dactylus with 2 (anterior and posterior) subterminally inserting claws and posterior to both claws with elongated expansion, proximodorsally to claws with 2–3 sensillae, exceeding the distal tip in length. Pereopod 3 most robust, strongly setiferous, ischium extended dorsally, with row of long setae, with 1–2 apical setae strongly pronounced (spine-like, thickened, bent), merus distally extended; orientation of propodus twisted 180° along proximo-distal axis compared to pereopods 1–2, hence, propodus and dactylus bent in dorsal direction instead of ventral. Pereopod 4 shortest, bent laterally at mero-carpal articulation, directed in lateral or laterodorsal position. Posterior pereopod articles elongate subcylindrical. Pereopodal coxae inserted lateroventrally.

Coxae 1–3 inserted at anteriolateral margins of pereonites, coxae 4 inserted medially, coxae 5–7 inserted under posterolateral protrusions.

Sternite of pleonite 1 distinguishable; pleopodal articulations merged together at anterior margin of branchial cavity; anus subterminally, separated from branchial cavity in longitudinal ventrocaudal excavation stretching from branchial cavity to pleotelson apex. Female operculum oblong, distally with long pappose setae covering anal chamber. Uropods with 1-, 2- or many articles, elongated, terminally articulated, uniramous. Pleotelson with a pair of statocysts.

#### Genus Macrostylis

G.O. Sars, 1864

Macrostylis Sars 1864, p.13; 1899, p. 120; Beddard 1886b, p. 173; Hansen 1916, p. 75; Barnard 1920, p. 411; Wolff 1956, p. 99–106; 1962, p. 91–93; Menzies 1962, p. 127–133; Birstein 1963a, p. 95–106; Mezhov 1988, p. 60–69; Brandt 1992, p. 74–78; Vey & Brix 2009, p. 358
Vana Meinert 1890, p. 195
Desmostylis Brandt 1992, p.70, Figs 11–13

##### Composition.

See Table 2.

**Table 2.** Composition and distribution of Macrostylidae Hansen, 1916.

Taxon		Locality	Depth (m)
Macrostylis G. O. Sars, 1864			
	Macrostylis abyssalis Brandt, 2004	S Atlantic, Angola Basin	5389
	Macrostylis abyssicola Hansen, 1916	NW Atlantic, Davis Strait	698–3921
	Macrostylis affinis Birstein, 1963	NW Pacific	4690–5554
	Macrostylis amplinexa Mezhov, 1989b	Indian Ocean	2385–4221
	Macrostylis angolensis Brandt, 2004	SE Atlantic, Angola Basin	5395
	Macrostylis angulata Mezhov, 1999	NE Atlantic	5420–6051
	Macrostylis antennamagna sp. n.	Southern Ocean, NW Weddell Sea	4698–4760
	Macrostylis belyaevi Mezhov, 1989a	N Pacific	8540–8780
	Macrostylis bifurcatus Menzies, 1962	SE Atlantic	4588–4960
	Macrostylis bipunctatus Menzies, 1962	SW Atlantic	3954–5024
	Macrostylis birsteini Mezhov, 1993	S Pacific	1200
	Macrostylis capito Mezhov, 1989b	Indian Ocean	2218–4737
	Macrostylis caribbicus Menzies, 1962	W Atlantic, Caribbean, Columbia	2875–941
	Macrostylis carinifera carinifera Mezhov, 1988	Indian Ocean	3074–4458
	Macrostylis carinifera dilatata Mezhov, 1988	Indian Ocean	2540
	Macrostylis cerritus Vey & Brix, 2009	Southern Ocean, Weddell Sea	2149
	Macrostylis compactus Birstein, 1963	W Pacific, Bougainville Trench	6920–7954
	Macrostylis confinis Mezhov, 2003	NW Indian Ocean	3617
	Macrostylis curticornis Birstein, 1963	NW Pacific	5680–6670
	Macrostylis dellacrocei Aydogan, Wägele & Park, 2000	SE Pacific, Atacama Trench	7800
	Macrostylis diatona Mezhov, 2004	E Indian Ocean	6433
	Macrostylis elongata Hansen, 1916	N Atlantic, Iceland	1591
	Macrostylis emarginata Mezhov, 2000	N Atlantic	5420
	Macrostylis expolita Mezhov, 2003b	N Indian Ocean, Arabian Sea	2478–2519
	Macrostylis foveata Mezhov, 2000	W Atlantic, Puerto Rico Trench	5060–6650
	Macrostylis fragosa Mezhov, 2004	E Indian Ocean	5410
	Macrostylis galatheae Wolff, 1956	W Pacific, Philippine Trench	8440–10000
	Macrostylis gerdesi (Brandt, 2002) comb. n.	Southern Ocean, Maud Rise	238
	Macrostylis gestuosa Mezhov, 1993	W Pacific	5526
	Macrostylis grandis Birstein, 1970	NW Pacific, Kurile-Kamchatka Trench	7265–7295
	Macrostylis hadalis Wolff, 1956	W Pacific, Banda Trench	7270
	Macrostylis hirsuticaudis Menzies, 1962	SE Atlantic	2997
	Macrostylis lacunosa Mezhov, 2003b	N Indian Ocean	4706–4737
	Macrostylis latifrons Beddard, 1886	N Pacific	3749
	Macrostylis latiuscula Mezhov; 2003b	Central Indian Ocean	4730–4808
	Macrostylis longifera Menzies & George, 1972	E Pacific, Peru-Chile Trench	4823–6134
	Macrostylis longipedis Brandt, 2004	S Atlantic, Angola Basin	5389
	Macrostylis longipes Hansen, 1916	N Atlantic, Iceland	325–1412
	Macrostylis longiremis (Meinert, 1890)	N Atlantic, Skagerrak	149–228
	Macrostylis longispinis Brandt, 2004	S Atlantic, Angola Basin	5415
	Macrostylis longissima Mezhov, 1981	N Central Pacific	6043–6051
	Macrostylis longiuscula Mezhov, 1981	N Central Pacific	4400
	Macrostylis longula Birstein, 1970	N Pacific	5005–5045
	Macrostylis magnifica Wolff, 1962	NW Atlantic, Davis Strait	3521
	Macrostylis mariana Mezhov, 1993	W Pacific	10223–10730
	Macrostylis medioxima Mezhov, 2003b	NW Indian Ocean	4458
	Macrostylis meteorae Brandt, 2004	S Atlantic, Angola Basin	5387–5390
	Macrostylis minuscularia Mezhov, 2003b	NW Indian Ocean	3617
	Macrostylis minutus Menzies, 1962	W Atlantic, Puerto Rico Trench	5163–5494
	Macrostylis obscurus (Brandt, 1992) comb. n.	Southern Ocean, Weddell Sea	4335
	Macrostylis ovata Birstein, 1970	NW Pacific, Kurile-Kamchatka Trench	6435–6710
	Macrostylis pectorosa Mezhov, 2004	E Indian Ocean	2807
	Macrostylis polaris Malyutina & Kussakin, 1996	Arctic Ocean	325–400
	Macrostylis porrecta Mezhov, 1988	Indian Ocean	6433
	Macrostylis profundissima Birstein, 1970	NW Pacific, Kurile-Kamchatka Trench	8185–9530
	Macrostylis prolixa Mezhov, 2003a	NW Indian Ocean	4458
	Macrostylis pumicosa Mezhov, 2004	E Indian Ocean	2917
	Macrostylis quadratura Birstein, 1970	NW Pacific, Kurile-Kamchatka Trench	3175–3250
	Macrostylis rectangulata Mezhov, 1989b	Indian Ocean	5220
	Macrostylis reticulata Birstein, 1963	NW Pacific	5502
	Macrostylis robusta Brandt, 2004	S Atlantic, Angola Basin	5497–5398
	Macrostylis sarsi Brandt, 1992b	Southern Ocean, Weddell Sea	4335
	Macrostylis sensitiva Birstein, 1970	NW Pacific, Kurile-Kamchatka Trench	5005–5100
	Macrostylis setifer Menzies, 1962	W Atlantic, Puerto-Rico Trench	5477–5494
	Macrostylis setulosa Mezhov, 1992	Southern Ocean, Scotia Sea	757–2705
	Macrostylis spiniceps Barnard, 1920	S Atlantic, South Africa	1280
	Macrostylis spinifera Sars, 1864	N Atlantic, Norwegian Sea	27–1710
	Macrostylis squalida Mezhov, 2000	Central Atlantic, Romanche Trench	6380–6430
	Macrostylis strigosa Mezhov, 1999	NE Atlantic	5420
	Macrostylis subinermis Hansen, 1916	N Atlantic, Norwegian Sea	830–3474
	Macrostylis truncatex Menzies, 1962	NW Atlantic	3950–3963
	Macrostylis tumulosa Mezhov, 1989	W Pacific, Izu-Bonin Trench	8900
	Macrostylis uniformis sp. n.	Southern Ocean, Weddell Sea	4651–4975
	Macrostylis urceolata Mezhov, 1989b	Indian Ocean	2596
	Macrostylis vemae Menzies, 1962	W Atlantic, Puerto-Rico Trench	5410–5684
	Macrostylis vigorata Mezhov, 1999	NE Atlantic	2655–2667
	Macrostylis vinogradovae Mezhov, 1992	Southern Ocean, Weddell Sea	2705–4335
	Macrostylis viriosa Mezhov, 1999	NE Atlantic	4050
	Macrostylis vitjazi Birstein, 1963	W Pacific, Bougainville Trench	6920–7954
	Macrostylis wolffi Mezhov, 1988	Indian Ocean	2385–3717
	Macrostylis zenkevitchi Birstein, 1963	NW Pacific	4690–6135

##### Type species.

Macrostylis spinifera Sars, 1864

##### Diagnosis.

As of the family.

##### Remarks.

Some characters are absent or poorly illustrated in original descriptions and could not be analysed thoroughly during this study. Therefore these have not been included in the family diagnosis (e.g. presence of exopod of pleopod 5, setation patterns and setal substructures).

Following the original description (Brandt 1992), Desmostylis can be separated from Macrostylis by the following characters: absence of dorsal triangular expansion on the pereopod 3 ischium, lack of dactylus on pereopod 4 and of claws on pereopods 5–7. However, after comparisons of different species of Macrostylis, and re-examination of the holotype of Desmostylis obscurus Brandt, 1992 these characters have been found not to be delimitating Desmostylis from Macrostylis. Shape and extension of pereopod 3 ischium varies greatly between species of Macrostylis. For example Macrostylis galatheae Wolff, 1956 (p. 101, Fig. 17) has a strong and acute extension and no extension is present in Macrostylis abyssalis Brandt, 2004 (p. 28, Fig. 15), very similar to Desmostylis obscurus. However, another described species of Desmostylis, Desmostylis gerdesi Brandt, 2002 shows a strongly fossorial pereopod 3 bearing a prominent dorsal extension on the ischium. Thus, the condition of this character in Desmostylis lies within the interspecific range of variation in Macrostylis. Therefore, this character is not usable to maintain the genus Desmostylis.

The absence of a pereopod 4 dactylus was another generic character of Desmostylis. However, the dactyli in Desmostylis obscurus are not absent but have been overseen in the original description and they were distorted in the pereopod illustrated of the holotype. The value of differences in setal counts or occurrence of types of setae on pereopod 3 ischium for discriminating between two genera is not known. The genus Desmostylis was erected on basis of a juvenile specimen. Using ontogenetically variable characters of a juvenile type specimen to erect a new genus is problematic.

Dactylar setae fit the definition of claws (Wilson 1989): terminal/subterminal modified setae on pereopodal dactyli with pronounced sclerotisation and a sharp tip. This definition was to the authors’ knowledge not narrowed since then. Taking into account the incomplete documentation of dactylar claws in the literature on the one hand, and insufficient knowledge about plasticity of setae and their substructures on the other, a differentiation of genera exclusively based on such characters seems problematic. Due to the above listed reasons we consider the genus Desmostylis synonymous with Macrostylis.

##### Macrostylis uniformis sp. n.

urn:lsid:zoobank.org:act:5105DA6E-E793-42B6-A5D7-A4D9F8C5933A

Figs 3 8 

###### Material examined.

Holotype. Preparatory female, 4.6 mm long, ZMH (K-42172). South-eastern Weddell Sea southwest of Maud Rise; station ANTXXII-3 59-5 (ANDEEP III; 67°29.81’S; 000°00.23’W); 4651 m depth. Paratypes. 1 paratype, ovigerous female, damaged, ZMH (K-42173) from type locality; 1 paratype female without oostegites fixed for SEM, 3.5 mm, ZMH (K-32174) northern Weddell Sea, station 137 (ANDEEP II; 63°45.00’S; 033°47.81’W), 4975 m depth. For further material examined for comparison see: Table 1.

###### Diagnosis.

Cephalothorax almost semicircular, little longer than wide with no transverse ridge on frons; antenna 1 minute, incl. aesthetascs not reaching article 4 of antenna 2; mandible with blunt pars incisiva, left lacinia mobilis spiniform, integrated into spine row; dorsal extension of ischium of pereopod 3 positioned much more distally, on apex with 2 conspicious setae, 1 bent robust and spiniform unequally bifid seta proximally, and another more straight and less robust unequally bifid seta distally; posterolateral corners of posterior pereonites rounded; pleotelson compact, not constricted anteriorly of uropod articulations; uropod endopod of half the length of protopod.

###### Description of holotype female.

**Figure 3.**
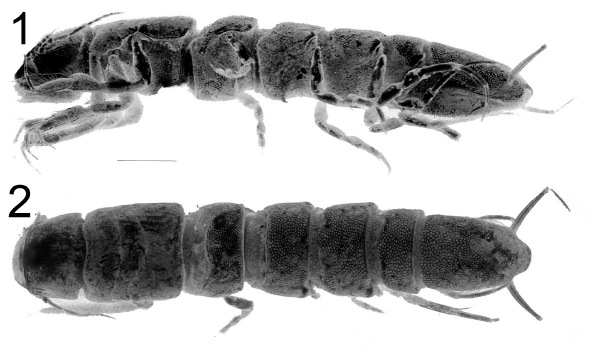
Macrostylis uniformis sp. n. (Holotype ♀, ZMH (K-42172)) digital stack photograph of female holotype; **1** lateroventral view; stained with methylene green H2O solution; colours inverted, greyscale; scale bar = 0.5 mm **2** dorsal view; stained with methylene green H2O solution; colours inverted, greyscale; scale bar = 0.5 mm.

**Figure 4.**
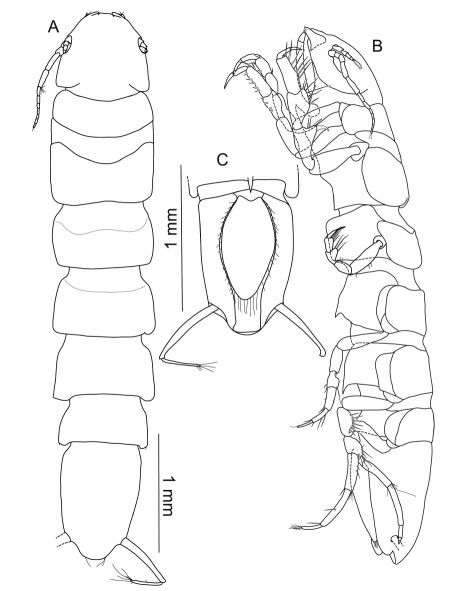
Macrostylis uniformis sp. n. (Holotype ♀, ZMH (K-42172)) **A** habitus (dorsal view, pereopods and left uropod omitted) **B** habitus (lateral view), uropods omitted **C** pleotelson (ventral view).

Body (Figs 3–4) elongate, 5.3 times longer than wide; maximal body width in pereonite 3 1.2 times maximal width of pleotelson; pereonites 1–5 about the same width, gradually narrowing from pereonite 6 towards pleotelson. Surface of tergites, sternites and operculum bearing comb-like structures, which can be worn off due to abrasion to a smooth surface in exposed areas (e.g. cephalothorax, pereonite 3); posterolateral setae only in pereonite 7, otherwise lacking or broken off; no sockets found in SEM (probably due to dirt on cuticle). Cephalothorax free, almost semicircular with maximal width at posterolateral margin, length 1.1 times maximal width and 0.2 times total body length; 0.9 times width of pereonite 1; no transverse ridge on frons.

Fossosome 1.1 times longer than wide, laterally slightly convex, median length: pereonite 1 about 1.4 times longer than pereonite 2, as long as pereonite 3; lateral length: pereonite 1 1.3 times as long as pereonite 2 and 0.5 times as long as pereonite 3; pereonite with prominent anteroventral spine and pereonite 3 with very small posteroventral spine. Pereonites 4–5 of same length and width, 1.6 times wider than long; pereonite 4 laterally convex, maximal width amid segment. Pereonites 5–7 posterolateral corners tapering, with tiny simple apical setae; pereonites with short posterior ventral spines. Pereonite 6 0.8 times pereonite 4 length, 1.7 times wider than long. Pereonite 7 0.7 pereonite 4 length, 1.9 times wider than long; Pleotelson 1.5 times longer than wide, length about 0.2 times total body length, as long as fossosome and as long as pereonites 5 and 6 together; laterally convex, slightly narrowing towards uropodal articulations with no constriction; apex bluntly rounded with several long setae; compared to rest of body with strongest sculpturation of cuticle; cuticle not translucent, dorsal organ not visible; slot-like apertures in dorsal cuticle not present; pleopodal chamber maximal opening width 0.6 times maximal pleotelson width; longitudinal excavation minimal width about 0.3 times max pleotelson width.

**Figure 5.**
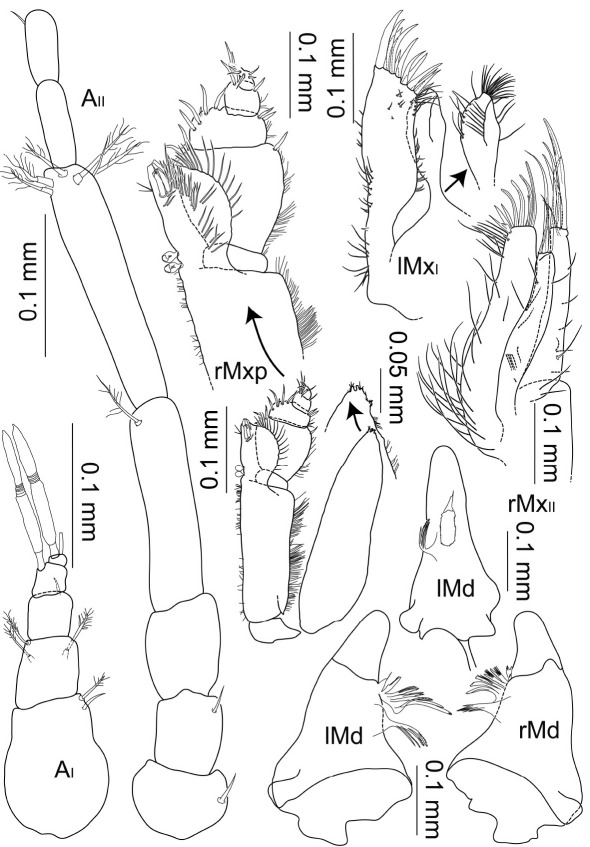
Macrostylis uniformis sp.n. (Holotype ♀, ZMH (K-42172)) antenna 1; antenna 2 (flagella broken off); left mandible (dorsal and medial view); right mandible (dorsal view); right maxilliped (dorsal view, some setae omitted); right maxilliped (enlarged endite and palp), right maxilla 1 (with inner endite illustrated separately), right maxilla 2.

Antenna 1 (Figs 3–5) of 5 articles, 0.25 times fossosome median length; 2,6 times longer than wide; articles gradually decreasing in size and length-width ratio towards distal end, relative length ratios: 1:0.5:0.3:0.2:0.1; article 1 length 1.4 times of width, 50% of total antenna 1 length, article 5 as long as wide, less than 0.1 times total antenna 1 length; articles 4 and 5 with 1 aesthetasc each; articles 1 and 2 with distal broom setae; simple setae on distal margins of articles 2 and 3, 1 seta on article 5.

Antenna 2 (Figs 3–5) basal five articles reaching the posterior end of cephalothorax; flagellum reaching the anterior margin of pereopod 2 basis when directed posteriorly; article 1 broadest, 1.4 times wider than long, article 2 1.2 times longer than wide and article 3 1.4 times longer than wide, article 4 little narrower than articles 1–3, 4 times longer than wide, article 5 longest 1.1 times article 4, 4.5 times longer than wide; several broom setae distally on basal articles, most on article 5; seven flagellar articles, width about 0.5 times article 5 width.

Mandible (Fig. 5) gradually narrowing towards pars incisiva; pars incisiva blunt and rounded, without teeth; left lacinia mobilis spine-like, with subtriangular basis in dorsal and medial view and 1 small spine on apex, little shorter than adjacent spine row; right lacinia mobilis tiny with several short spine-like projections; spine row of 5–7 fanned spines, more in left mandible, partially serrated at tips and along proximal margin, especially more proximal ones; pars molaris shorter than adjacent spines of spine row, apex oriented proximally.

Maxilla 1 (Fig. 5) inner endite shorter and more slender than outer one, terminally spatulate; dense accumulation of simple setae around distal apex, along a dorsal ridge as well as along medial and lateral margins with setae; outer endite broad, narrowing in the most distal quarter, with numerous setae of different lengths on lateral and medial margins, numerous setae of different lengths around distomedial corner 12 robust setae, some two-sided serrate, on distal margin.

Maxilla 2 (Fig. 5) inner and outer endites of similar width, equally projecting distally; medial endite thinner and shorter; along proximedial margin of inner endite more than 15 long simple setae of less than half the length of inner endite, an accumulation of about 10 small and intermediate simple setae distolaterally, on distal margin 7 strong setae, some heavily denticulate, medial endite with few simple setae along lateral and medial margins, distally with 3 simple setae of different lengths, longest seta less than half as long as the medial endite; outer endite with simple setae around lateral margin, distally with four robust setae of different lengths the most lateral one longest and one-sided denticulate.

**Figure 6.**
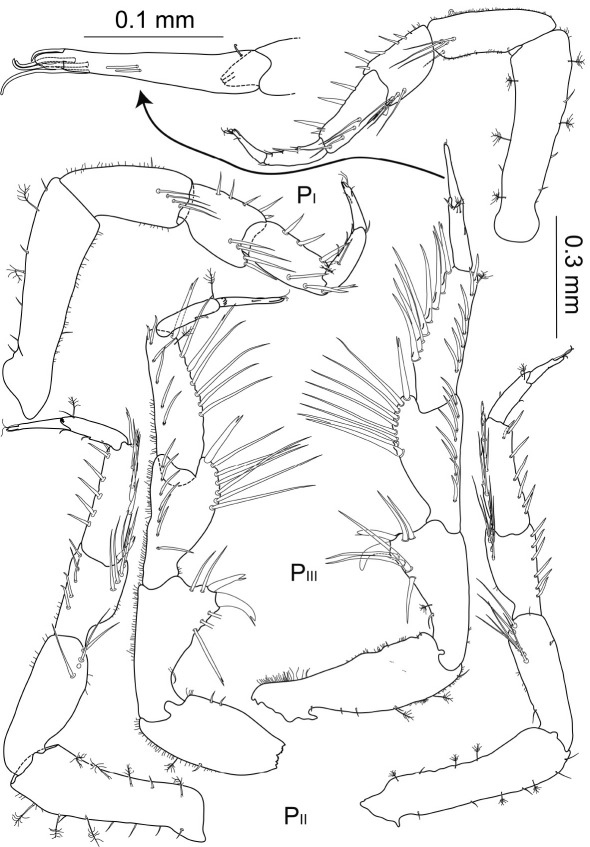
Macrostylis uniformis sp. n. (♀) pereopods 1–3 (P_I–III_; 0.3 mm scale) of preparatory female (holotype, ZMH (K-42172); left side) and ovigerous female (paratype, ZMH (K-42173, right side); with dactylus of pereopod 3 enlarged (0.1 mm scale).

Maxilliped (Fig. 6) epipod 3.6 times longer than wide, distally narrowing to multiple small tips, with tiny setae or setules, laterally concave, reaching midlength of palp article 3; endite medially thickening, proximomedially with two coupling hooks, distally with heavily sclerotized and denticulate tooth-like setae and fine dense setae; row of setae along rounded distolateral margin, lateral margins of basis and palp articles 1–3 with rows of thin setae; article 2 largest and longest, 3.5 times longer than article 1 and 2.3 times longer than article 3; articles 4 and 5 distomedially and distolaterally with medially scaled and distally pappose sensillae: 3 medially and 1 laterally on article 2, 5 medially and 2 laterally on article 3, 3 medially and 1 laterally on article 4, article 5 with 6 such setae terminally and subterminally; basis including endite 0.9 times epipod length.

Anterior pereopods (Fig. 6) slightly increasing in length, pereopod 1 0.9 times length of pereopods 2 and 3 respectively; all articles covered with tiny setules of varying density of coverage.

Pereopod 1 relative length ratios: 1:0.6:0.4:0.4:0.3:0.2, basis with at least 3 broom setae and 1 short simple seta dorsally and 2 broom setae and row of 5 short setae ventrally, 3.6 times longer than wide; ischium 2.4 times longer than wide, with row of 4 long and slender setae distally, 1 seta on the opposite side, 1 short distally pappose sensilla ventrally, 1 short seta proximodorsally; merus compact, 1.4 times longer than wide, posteriorly on dorsal extension with row of 4 simple setae of different length and 1 bifurcate setae, along ventral margin 4 distally pappose setae and on distoventral extension 1 stout bifurcate seta; carpus 2.3 times longer than wide, distodorsally with row of 2 simple setae and 1 bifurcate seta most distally, 1 broom seta on distoventral margin, ventral margin with four distally fringed setae; propodus 3.4 times longer than wide, dorsally with 1 short setae, ventral side with 3 short sensillae, caudally with 1 long and slender seta; dactylus 3 times longer than wide, about 0.7 times carpus length, with 1 sensilla on dorsal and ventral side respectively.

Pereopod 2 1.1 times longer than pereopod 1; setation comparable to pereopod 1 with slight variations in length and numbers; relative length ratios: 1:0.7:0.5:0.6:0.4:0.3, basis 3.1 times longer than wide; ischium 2.6 times longer than wide; merus 1.8 times longer than wide, with row of 5 distally serrate setae; on the anterior side of distodorsal extension with 1 robust unequally bifid seta; carpus 2.8 times longer than wide, distodorsally with row of 5 long setae, first 4 of this setae distally serrate, most distal one bifurcate, 1 broom seta; propodus 4.5 times longer than wide, 1 broom seta and terminally expanded to subtriangular lobe dorsally with two notches at apex; dactylus 5 times longer than wide, anterior and posterior claw of about the same length, slender posterior extension clasping, reaching beyond claws.

Pereopod 3 1.1 times longer than pereopod 2, with bigger dorsal and ventral extensions and generally longer and more robust setae, relative length ratios: 1:0.7:0.7:0.8:0.3:0.3, basis damaged in holotype, 3.3 times longer than wide in paratype, with at least 2 broom setae and 3 short setae on ventral side, with 2 small setae on dorsal margin; ischium 1.8 times longer than wide, dorsal extension with concave flanks, with 3 serrate setae proximally and 2 distally of apex, on apex with 2 pronounced unequally bifid setae; proximal seta very robust and bent proximally; 1 seta bent towards proximal hollow of ischium articulation; merus about 2 times longer than wide with distodorsal and distoventral extensions, with row of 7 serrate setae dorsally, the most distal seta bifurcate and robust, along ventral margin with 7 distally pappose, fringed setae; carpus 3 times longer than wide, distodorsally with row of 7 serrate setae, most distal seta bifurcate, with 1 broom seta and 6 distally pappose, fringed setae ventrally; propodus 4.3 times longer than wide, distodorsally with 2 sensillae and 1 broom seta ventrally; dactylus long and slender, 6 times longer than wide, as long as carpus, with 1 proximal, 2 medial and 3 subterminal sensillae, claws as in pereopod 2.

**Figure 7.**
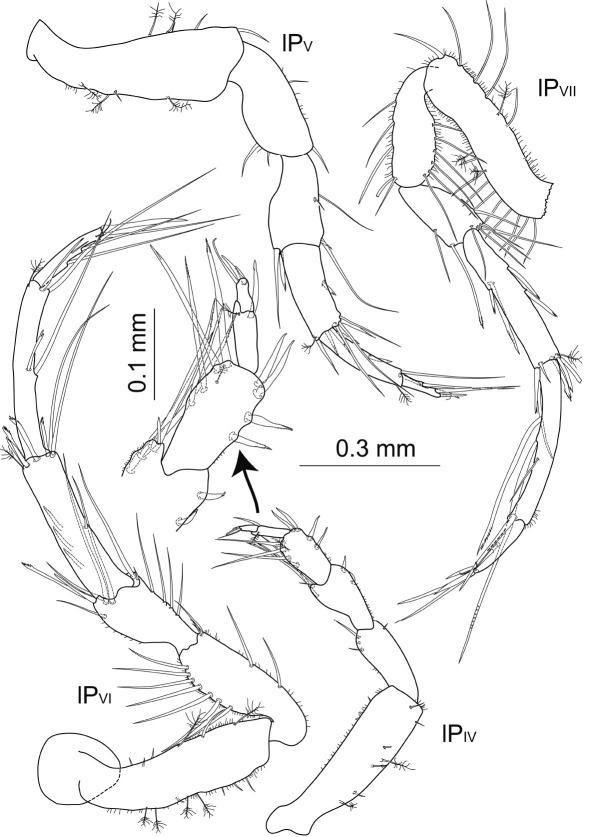
Macrostylis uniformis sp. n. (Holotype ♀, ZMH (K-42172)) pereopods 4–7 (P_IV–VII_; 0.3 mm scale); pereopod 4 dactylus enlarged (0.1 mm scale).

Posterior pereopods (Fig. 7; pereopods 6–7 of paratype missing) length ratios: 1:1.4:1.9:1.7.

Pereopod 4 about 0.6 times the length of pereopod 1, relative length ratios: 1:0.5:0.4:0.3:0.2:0.1, basis 3.7 times longer than wide with at least 4 broom and 2 short simple setae; ischium 2.9 times longer than wide, distodorsally with row of 3 setae and 1 tiny seta distoventrally; merus 1.9 times longer than wide, distodorsally expanded and with 4 setae of very different lengths, first 3 longer than carpus, serrate, the most distal one smaller, 2 distally pappose, fringed setae on ventral margin; carpus 2.2 times longer than wide, anterior-posteriorly flattened, and 3 times broader than propodus, dorsally extended with row of 4 long serrated, bifurcate setae reaching beyond dactylus tip, distodorsally with broom seta, on ventral margin with 4 distally pappose, fringed setae; propodus distally projecting into a subtriangular lobe, 3.5 times longer than wide, distoventrally with 1 long bifurcate and terminally fringed seta projecting beyond dactylus tip, 1 broom seta distally on dorsal side; dactylus of half propodus length, twice as long as wide, 1 short terminal claw, 1 subterminal claw of 0.5 times dactylus length, with 1 long thin seta subterminally and 2 sensillae.

Pereopod 5 0.9 times pereopod 1 length, relative length ratios: 1:0.6:0.5:0.5:0.4:0.2, basis broad, 2.9 times longer than wide with at least 3 broom setae dorsally, 2 broom setae and 3 small setae ventrally, 1 large simple seta distoventrally; ischium 2.3 times longer than wide, on dorsal and ventral side with 2 simple setae respectively; merus about 1.8 times longer than wide, distodorsally extending with 1 bifurcate and 1 long seta, with 2 tiny and 2 long setae on ventral side, longer seta longer than merus; carpus 2.6 times longer than wide,  wide, ventrally with 1 stout bifurcate seta, articulation to propodus surrounded by 5 bifurcate setae, 1 broom seta and ventrally with 3 long and slender simple setae, longest seta exceeding propodus in length; propodus 5 times longer than wide, with 1 short bifurcate seta ventrally, distally with 2 setae on ventral side, the longest more than 2 times dactylus length, 1 broom seta dorsally; dactylus 2 times longer than wide, half propodus length, with 2 setae ventrally of more than 2 times dactylus length, with 2 short claws, 0.7 times dactylus length; at least 2 thin subterminal sensillae.

Pereopod 6 1.2 times pereopod 1 length, relative length ratios: 1:0.6:0.4:0.7:0.7:0.4, basis 4.3 times longer than wide, dorsally with 3 short setae and 3 broom setae, ventrally with 4 broom setae and 4 short setae; ischium 2.7 times longer than wide, dorsally slightly projecting with row of 7 long setae, ventrally with 2 setae and distoventrally with a group of 3 simple setae of different lengths; merus short and broad, 1.8 times longer than wide, distodorsally extending with 6 setae, the longest exceeding carpus, some denticulate or bifurcate, with row of 6 setae on ventral side; carpus slender, 4.4 times longer than wide, dorsally with row of 3 short setae and 1 broom seta, 2 simple setae ventrally, distally 4 robust, bifurcate and one-sided serrate setae and 2 slender setae, longer than propodus; propodus 7.6 times longer than wide, along ventral margin with 2 groups of 1 short bifurcate and 1 slender seta each; dactylus 6 times longer than wide, with 2 small sensillae on anterior side, with 2 very long simple setae along ventral margin, with terminal claw as long as dactylus and 1 subterminal claw, 1.7 times longer than dactylus.

Pereopod 7 1.1 times longer than pereopod 1, relative length ratios: 1:0.6:0.4:0.8:0.9:0.4, basis 4.1 times longer than wide, dorsally with 9 simple setae as well as 2 broom setae, ventrally with 4 broom setae and 6 simple setae of different lengths as well as 1 broom seta; ischium 3.2 times longer than wide, dorsally with row of 6 long simple setae, ventrally with 5 simple setae; merus 2 times longer than wide, distodorsally extending with 6 long setae, on the ventral margin with 1 simple and 1 serrate seta; carpus slender, 5.7 times longer than wide, dorsally with 2 bifurcate setae and a broom seta distally, a group of serrate setae on ventral margin, mero-carpal articulation surrounded by 6 short, robust, bifurcate and one-sided serrate setae; propodus slender, 9 times longer than wide, 4 setae ventrally, the two proximal ones serrate and very long; dactylus 3.8 times longer than wide, with 1 intermediate and 1 long seta of 2 times dactylus length on ventral side, with 2 claws of different lengths and 1 sensilla on apex.

**Figure 8.**
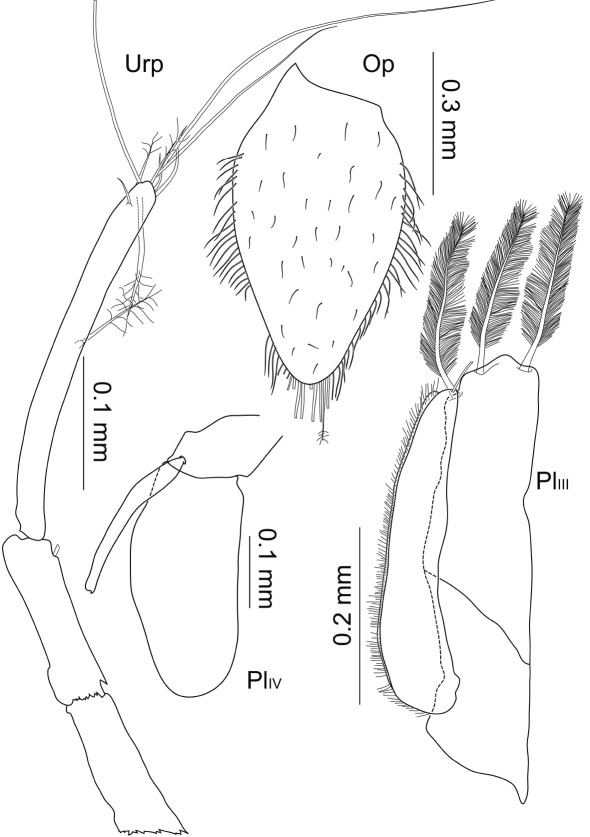
Macrostylis uniformis sp. n. (Holotype ♀, ZMH (K-42172)) operculum (Op; ventral view); pleopods 3–4 (Pl_III–PlIV_, ventral view); uropod (Urp).

**Figure 9.**
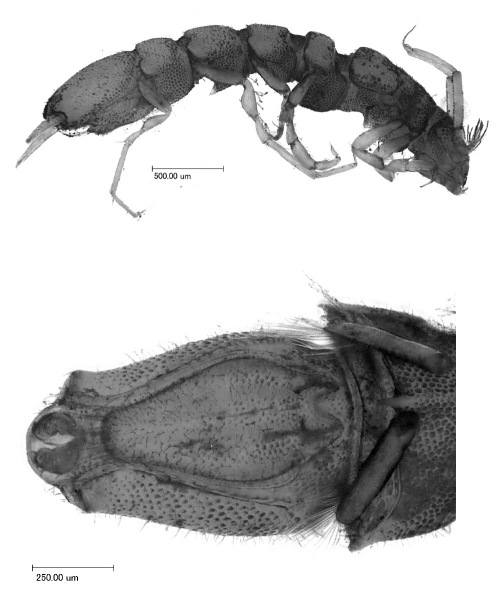
Top: Macrostylis antennamagna sp. n. (Holotype ♂, ZMH (K-42168)) digital stack photograph; stained with methylene green H2O solution, greyscale; below: Macrostylis antennamagna sp. n. (♀) digital stack photograph of pleotelson with operculum, ventrocaudal excavation and anus (ventral view).

Pleopods 2–4 (Fig. 8). Presence of pleopod 5 could not be clarified for both specimens. Relative length ratios: 1:0.8:0.6; operculum (Figs 5, 9) 0.7 times pleotelson length. Operculum covered ventrally with small setules and setae, along proximolateral margins with plumose setae with few short setules, distally with at least 8 pappose setae, several setae broken off and substructures not reconstructable. Pleopod 3 protopod constituting half of total length, rhomboid shape, endopod as wide as protopod, not considerably narrowing distally, with 3 distally plumose setae of 0.4 times pleopod 3 length, exopod 0.7 times total length, proximally about 0.7 times maximal width of endopod, distally narrowing, with 1 pronounced seta subterminally, row of numerous simple setae laterally. Pleopod 4 endopod long oval, 0.8 times total length; protopod as long as wide; exopod thin and long, 0.6 times total length and 7.7 times longer than wide, distal seta broken off and missing.

Uropod (Figs 3, 4, 8) of 2 articles, about half pleotelson length, protopod 5.5 times longer than wide, endopod 0.5 times protopod length, protopod with at least 1 broom seta distally, and endopod with at least 4 broom setae distally next to 3 very long and 3 short simple setae.

Male unknown.

###### Intraspecific variations in pereopods.

(Numerals in brackets and italics are variations in the ovigerous female, paratype).Pereopod 1 ischium 2.4 (3.2) times longer than wide, with row of 4 (3) setae distally. propodus 3.4 (2.8) times longer than wide; dactylus 3 (3.7) times longer than wide. Pereopod 2 basis 3.1 (3.7) times longer than wide; ischium 2.6 (3.2) times longer than wide; merus with row of 5 (6) setae; carpus distodorsally with row of 5 (6) long setae; propodus 4.5 (4) times longer than wide; dactylus 5 (5.5) times longer than wide.

Pereopod 3 ischium with 3 (4) setae proximally; merus with row of 7 (9) setae dorsally, along ventral margin with 7 (8) setae; carpus distodorsally with row of 7 (8) serrate setae, with 6 (7) setae ventrally. Pereopod 4 basis 3.7 (4.3) times longer than wide; ischium 2.9 (2.4) times longer than wide; merus 1.9 (2.4) times longer than wide; carpus 2.2 (2.8) times longer than wide; propodus 3.5 (3) times longer than wide. Pereopod 5 basis 2.9 (5) times longer than wide; ischium 2.3 (3) times longer than wide; carpus 2.6 (3.1) times longer than wide, propodus 5 (4.4) times longer than wide.

###### Etymology.

“Uniformis” is derived from the latin word for “uniform” as this species’ female on the first view resembles a most common macrostylid appearance and is hard to distinguish from other species.

###### Distribution.

Only known from the type locations: Southern Ocean, northern and south-eastern Weddell Sea, 4651–4975m depth.

###### Remarks.

Analysis of two specimens from different station reveals little variation. Differences in body shape and limb segments are usually too subtle to be detected in visual inspection. Variation in setal count tends to be allometric in the pereopods (compare Hessler 1970 for Desmosomatidae). Variation was observed in pereopod 3 in setal counts on ischium, merus and carpus, but none in length-width ratios. Setal variation also occurs in pereopod 1 ischium as well as pereopod 2 merus and carpus. In all cases, the number of setae is increased by one or two per row in the female paratype. In pereopods 1, 2 and 5 length-width ratios of all articles are increased or identical in the ovigerous female except for the propodus. The strong variation in pereopod 4 has to be treated carefully, as the articles are flattened and in the appendage contortions along the proximo-distal axis limit comparability between both specimens.

In the shape of the cephalothorax and lateral pleonite borders as well as the pleotelson apex Macrostylis uniformis sp. n. closely resembles that of Macrostylis hadalis Wolff, 1956, Macrostylis zenkevitchi Birstein, 1963 and Macrostylis longifera Menzies & George, 1972. Macrostylis uniformis sp. n. also shares the small subtriangular lacinia mobilis and the minute pars molaris with Macrostylis hadalis. They can be separated by bicusp and acute pars incisiva in Macrostylis hadalis (blunt and rounded in Macrostylis uniformis sp. n.) and the smaller relative length of the antenna 2 compared to the antenna 1 in Macrostylis hadalis. Macrostylis zenkevitchi has more acute posterolateral corners of the posterior pereonites than found in Macrostylis uniformis sp. n. The mandible of Macrostylis zenkenvitchi has incisors with 3 blunt teeth and a strong lacinia mobilis (incisor without teeth, blunt and rounded, lacinia mobilis spine like and integrated into spine row in Macrostylis uniformis sp. n.). From Macrostylis longifera it can be distinguished by the pronounced posterolateral setae in pereonites 3–7 (no or tiny posterolateral setae in Macrostylis uniformis sp. n.) and in the stretched posterolateral protrusions of the posterior pereonites in Macrostylis longifera (protrusions subtle in Macrostylis uniformis sp. n.). The paratype female has damage on the pereon and exact measurements could not be taken. Nevertheless, length and width data from the anterior subsection indicate a high similarity in length and the ovigerous female being less wide in these pereonites.

No male specimen of this species could be identified and the male identity therefore remains unknown.

##### Macrostylis antennamagna sp. n.

urn:lsid:zoobank.org:act:5B09C3B7-6291-458D-BC5E-975BCBD39D80

Figs 9 18 

###### Material examined.

Holotype. Adult male, 3.4 mm, ZMH (K-42168), Southern Ocean, North-western Weddell-Enderby Abyssal Plain, south of the South Orkney Islands, station 110-8 (ANDEEP III), 64°59.20’S; 043°002.05’W, 4698 m depth. Paratypes. 1 preparatory female, 4.7 mm, ZMH (K-42169) and 1 adult male ZMH (K-42169) from type locality; 1 male , 3.1 mm, ZMH (K-42171); 1 male fixed for SEM, 2.8 mm, ZMH (K-42172) Southern Ocean, northern Weddell Sea abyssal plain, south of the Endurance ridge, station 138-6 (ANDEEP II), 64°1.67’S; 39°7.68’W, 4760 m depth. For further material examined for comparison see Table 1.

###### Diagnosis.

Cephalothorax semicircular, antenna 2 long, basal 5 articles pronounced, flagellum reaching to the posterior end of pereonite 3 when bent backwards, little shorter in female; mandible flat, dorsoventrally constricted proximally to laciniae; pars incisiva without pronounced or sharp teeth but bump-like structures; dominant setae on pereopods of bifurcate type; setation pattern on male pleopod 1 mostly not symmetrical, distolateral lobes of pleopod 1 projecting less distally than medial lobes, bent laterally (~90°) and ventrally, medial lobes rounded, each with 7 setae.

###### Description holotype male.

**Figure 10.**
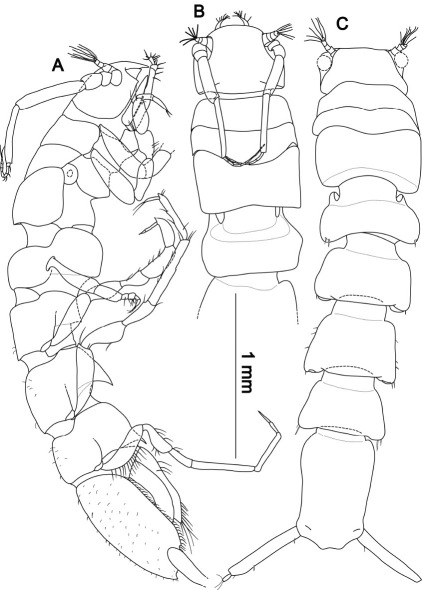
Macrostylis antennamagna sp. n. (Holotype ♂, ZMH (K-42168)) **A** lateral view, pereopod 3 broken off, uropods omitted **B** dorsal view anterior **C** dorsal view, AII and pereopods omitted, anterior part flexed.

Body (Figs 9, 10) elongate, 5.3 times longer than wide; maximal body width in pereonites 2 and 3, maximal pereonite width 1.5 times maximal width of pleotelson; pereonites 1–3 about the same width, from pereonite 3 towards pleotelson slightly but gradually narrowing, pereonite 7 0.8 times as wide as pereonite 3. Surface of body (Fig. 10) bearing comb-like structures which can be worn off in exposed areas, e.g. posterolateral protrusions, cephalothorax or dorsal surface of pereonite 3; numerous small simple setae and setules on general cuticle; pereonites 3–7 unsymmetrical with respect to number and length of 1–2 posterolateral setae. Cephalothorax almost semicircular, length 0.7 times maximal width posteriorly to antenna 2 articulation and less than 0.2 times total body length; width 0.9 times pereonite 1 width; transverse ridge on frons not recognized. Fossosome 0.2 times total body length; as long as wide, laterally slightly convex; median legth: 1:1:1.3; frontal spine in sternite of pereonite 1, dorsoventral constrictions close to the segment borders of pereonites 2–3. Posterior pereonites anteriorly strongly constricted; anterior margins overlapped by preceding tergite; relative medial lengths of pereonites 4–7: 1:1.2:1.4:1.2; laterally concave. Posterolateral parts of tergites slightly tapered backwards; pereonites 5–7 with ventral spines directed posteriorly. Pleotelson 0.2 times body length; about 1.7 times longer than wide, laterally concave, narrowing towards a constriction anterior to uropodal articulations; caudal apex concave surrounded by setules; compared to rest of body, most prominent sculpturation of cuticle, shingle-like appearance; cuticle slightly translucent, statocysts visible; slot-like apertures in dorsal cuticle; breathing cavity (Fig. 15) maximal opening width 0.7 times maximal pleotelson width, narrowest width of longitudinal excavation 0.2 times maximal pleotelson width.

**Figure 11.**
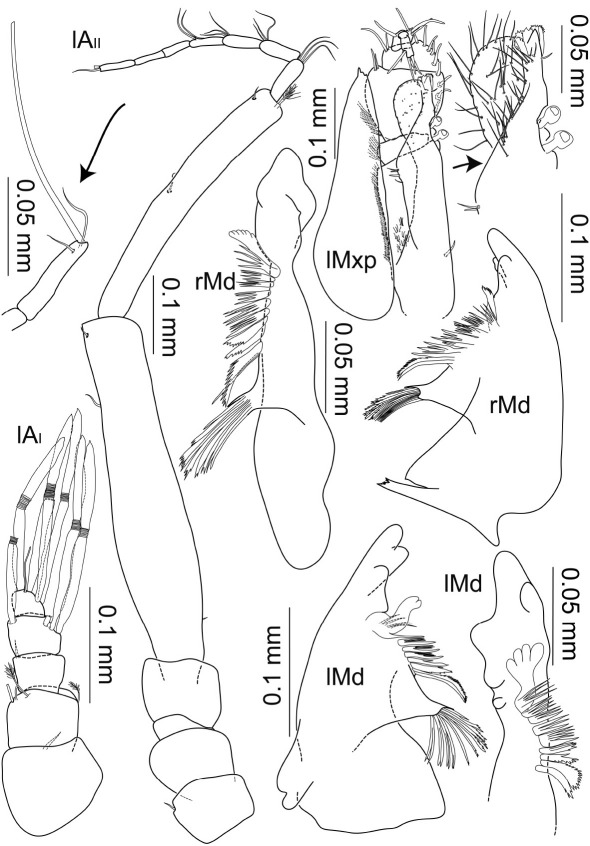
Macrostylis antennamagna sp. n. (Holotype ♂, ZMH (K-42168)) left antenna 1; left antenna 2; right antenna 2 enlarged last flagella article; left mandible (dorsal and medial view); right mandible (dorsal and medial view); left maxilliped (dorsal view, some setae omitted); left maxilliped (enlarged endite).

Antenna 1 (Figs 10, 11) with 5 articles, small, barely reaching halfway to posterior margin of cephalothorax when directed backwards; length 2.6 times article 1 width; all articles about as long as wide, gradually decreasing in size towards distally; fifth article 0.1 times total length; at least 4 aesthetascs on each of articles 4 and 5, some broken off; broom setae around distal margin of article 2; some distally fringed sensillae on distal margins of articles 1–3; with 1 seta distally on article 5, next to aesthetascs.

Antenna 2 (Figs 10, 11) relatively long and broad, peduncle reaching to the posterior end of pereonite 2 and flagellum reaching to the posterior end of pereonite 3; each of articles 1–3 1.4 times longer than wide; article 4 longest, length 0.3 times total length, as wide as articles 1–3; article 5 0.8 times article 4 length, about 0.4 times article 4 width; flagellum of 7 articles; all 5 basal articles with 1–several distal broom setae (not shown in illustration) and some simple setae; tubular setae with large apical pores distally on flagellar articles 1–4 ans 7, articles 1–3 with 3 setae each, articles 4 and 7 with 2; article 7 with long simple distal setae, 3 times longer than article.

Mandible (Fig 11) flat, dorsoventrally constricted proximally to lacinia; pars incisiva without teeth, but bump-like structures, left mandible with 1 terminal, 1 ventral and 2 dorsal cusps, right mandible with a centered lobe-like cusp; left lacinia mobilis longer and more robust than right lacinia mobilis, with 4 strong blunt teeth, 1.5 times longer than following spines of spine row; right lacinia mobilis twice as long as following spines of spine row, with 6 teeth, more acute than in left lacinia mobilis, dorsoventrally arranged, with ventral teeth projecting most distally; spine row of about 8 spines with multiple cusps, partially serrated; Maxilla 1 and 2: see description of female paratype.

Maxilliped (Fig. 11) epipod oblong-subtriangular, distally narrowing to multiple small tips, distolaterally concave, without any setae or setules, reaching distal end of palp article 2; endite with 2 coupling hooks, densely covered with fine but rather long simple setae, medially broadening, more proximally and dorsally forming lobe-like protrusion with 4 setae on lateral blunt apex, together constituting a sheath in which the epipod rests; lateral margins of basis, and palp articles 1–2 with row of small setae, basis and epipod subequal in length; palp article 2 3 times longer than article 1, articles 2–4 distomedially and distolaterally with medially scaled and distally pappose (fringed) sensillae, article 5 with the same setae terminally and subterminally.

**Figure 12.**
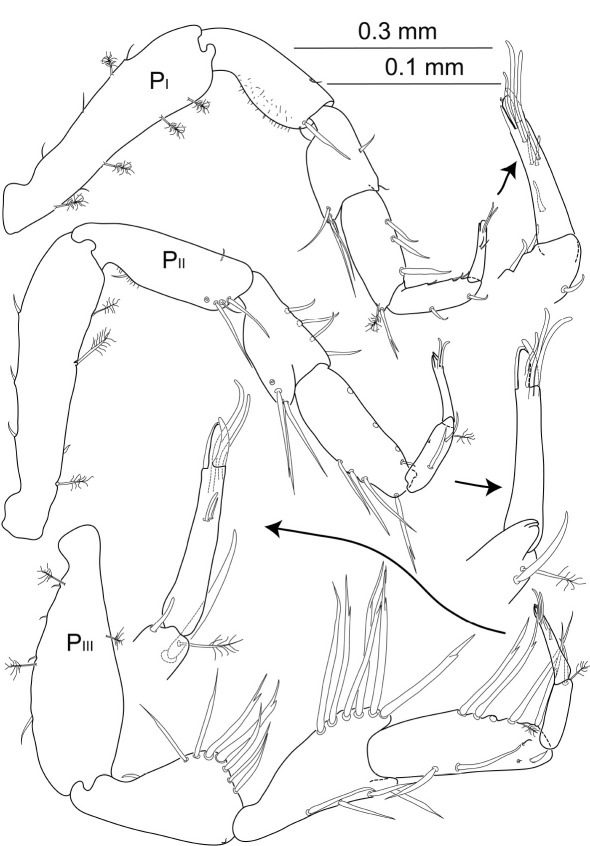
Macrostylis antennamagna sp. n. (Holotype ♂, ZMH (K-42168)) pereopods 1–3 (P_I–III_; 0.3 mm scale) with dactyli enlarged (0.1 mm scale).

Anterior pereopods (Fig. 12) length ratios: 1:1.1:1.1, all articles covered with tiny setules of varying density of coverage.

Pereopod 1 relative length ratios: 1:0.5:0.4:0.3:0.4:0.2, basis 3.8 times longer than wide, with 4 broom setae and 1 short simple seta on dorsal side, 3 short simple setae and 1 broom seta ventrally,; ischium 2.3 times longer than wide, with 2 setae distodorsally and 1 small seta distoventrally; merus almost 1.4 times longer than wide, with distodorsal extension bearing 4 unequally long setae; carpus 2.4 times longer than wide, distodorsally with 1 bifurcate seta and 1 broom seta, 3 setae ventrally; propodus 3.5 times longer than wide, dorsally with 2 short setae, ventral side with 3 sensillae, terminally expanded to a subtriangular lobe; dactylus 4.6 times longer than wide, 0.7 times the length of carpus, with 7 sensillae of different lengths.

Pereopod 2 relative length ratios: 1:0.6:0.3:0.5:0.2:0.2, basis with 3 broom setae dorsally and row of short setae ventrally, 4.2 times longer than wide; ischium 3.1 times longer than wide, with 1 small seta bent backwards proximodorsally, row of 4 long simple setae distodorsally and 1 small seta distoventrally; merus 1.5 times longer than wide with distodorsal extension bearing row of 4 long simple setae; carpus 2.6 times longer than wide, distodorsally with row of 4 long setae, most distal one bifurcate, with 4 unequal simple setae ventrally; propodus 3 times longer than wide, dorsally with 2 simple setae, with 1 broom seta dorsally, terminally expanded to a subtriangular lobe; dactylus 5.2 times longer than wide, subterminally with 3 sensillae.

Pereopod 3, relative length ratios: 1:0.6:0.8:0.7:0.3:0.3, basis 2.9 times longer than wide with at least 3 broom setae, row of short setae on ventral side; ischium 1.8 times longer than wide, dorsal extension strongly expanded, sub-triangular, with 7 robust setae, apical 2 setae bifurcate, about as long as maximum ischium width, proximal 2 and distal 3 setae simple, but slightly increasing towards apex, proximodorsally 1 short seta, bent and directed towards proximal hollow of ischium articulation; merus twice as long as wide with weak distoventral extension and strong distodorsal extension bearing 6 bifurcate setae of similar length, 1.4 times maximal merus width, furcation not observed in most proximal seta of row, 4 simple setae ventrally; carpus 2.8 times longer than wide, distodorsally with 5 bifurcate setae and 1 broom seta, with 4 simple setae ventrally; propodus of 0.4 times carpus length, 3.6 times longer than wide, distoventrally with 1 simple and 1 broom seta, distodorsally with sensilla; dactylus 5.5 times longer than wide, 1.1 times carpus length, with 4 sensillae of different lengths arranged in 2 pairs on opposite sides.

**Figure 13.**
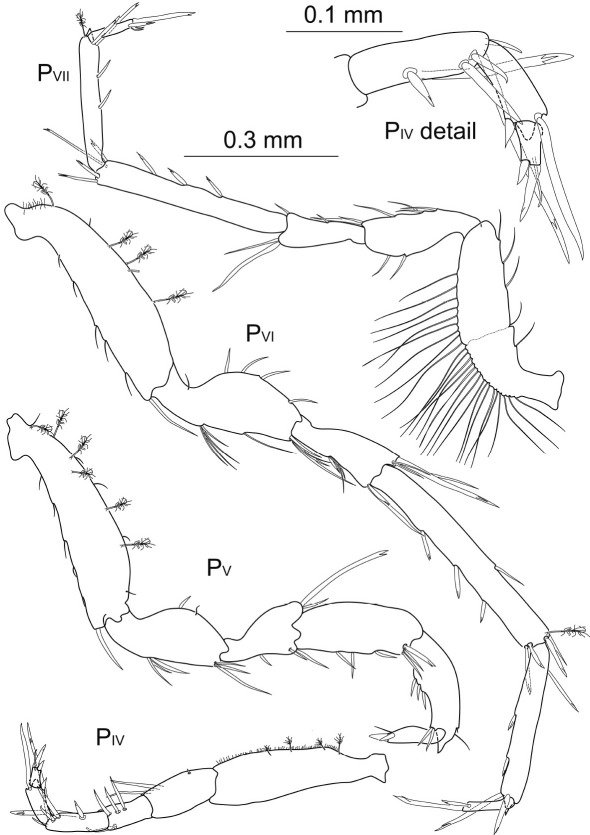
Macrostylis antennamagna sp. n. (Holotype ♂, ZMH (K-42168)) pereopods 4–7 (P_IV–VII_); pereopod 4 dactylus enlarged.

Posterior pereopods (Fig. 13) length ratios: 1:1.7:2.3:1.9.

Pereopod 4 0.6 times pereopod 2 length, relative length ratios: 1:0.4:0.3:0.3:0.2:0.1, basis 3.9 times longer than wide, with at least 4 broom setae; ischium twice as long as wide, with 2 short and 2 long setae; merus about 1.8 times longer than wide, row of 3 setae of same length as merus, most distal seta bifurcate, with 1 simple seta distally on opposite side of the article; carpus 3 times longer than wide, 1.3 times longer than merus, setation similar to merus but most proximal seta of row with bifurcation, and 1 very long bifurcate seta, almost reaching distal tip of propodus; with 1 very long bifurcate seta reaching the tip of the most distally reaching seta of propodus, projecting beyond all setae of dactylus; propodus distally projecting into a subtriangular lobe, 2.3 times longer than wide, distally with 1 long simple seta and 1 robust and acute seta; dactylus very small, twice as long as wide, about 0.4 times propodus length, with 1 terminal bifurcate claw of 1.3 times dactylus length and 1 subterminal claw of 0.5 times dactylus length, with 1 thin terminal seta of intermediate length.

Pereopod 5 as long as pereopod 2, relative length ratios: 1:0.6:0.4:0.6:0.5:0.2, basis 4.1 times longer than wide with at least 6 broom setae dorsally, 2 rows with 5 small, acute and distally directed setae respectively, arranged dorsally and ventrally, with 1 large simple seta distoventrally; ischium 2.3 times longer than wide, with 2 setae dorsally, 2 groups of 3 closely together articulating setae, 1 group medioventrally and 1 group distoventrally; merus 1.8 times longer than wide, distodorsally extending with 1 simple and 1 very long bifurcate seta of 1.1 times merus length, with 3 setae ventrally; carpus 2.5 times longer than wide, ventrally with 3 setae and distoventrally with 3 setae, the strongest of which is bifurcate; propodus 3 times longer than wide, distodorsally projecting into an acute subtriangular lobe, with 2 simple setae distodorsally and 2 on the opposite side, 3 setae ventrally, articulating closely together; dactylus small, 3.7 times longer than wide, of half propodus length, 2 claws, terminal claw of 0.4 times dactylus length, subterminal claw of 0.3 times the length of dactylus, with 1 thin subterminal seta ventrally.

Pereopod 6 1.3 times pereopod 2 length, relative length ratios: 1:0.6:0.4:1:0.7:0.2, basis 3.9 times longer than wide, setation as in pereopod 5 except no setae dorsally; ischium 2.4 times longer than wide, with 5 setae dorsally, 9 setae in 3 groups ventrally; merus about 2.3 times longer than wide, distodorsally extending with 4 unequal setae, the longest little longer than the article and bifurcate, 2 groups of setae ventrally; carpus elongated, as long as basis, 7.7 times longer than wide, dorsally with 1 seta and 1 broom seta, with 1 simple and 1 bifurcate seta distodorsally, ventrally with 3 robust bifurcate setae and distoventrally with a group of 3 bifurcate setae of varying lengths; propodus slender, 6.8 times longer than wide, distodorsally projecting into an acute subtriangular lobe, 2 bifurcate setae ventrally and 2 distoventrally, the longest one 1.5 times longer than dactylus; dactylus 5 times longer than wide, 0.3 times propodus length, with a terminal claw of 1.4 times dactylus length and a subterminal claw less than half as long.

Pereopod 7 1.1 times longer than pereopod 2, relative length ratios and setation similar to pereopod 6 except basis without broom setae, but dorsally with row of more than 25 simple setae of twice the length of basis width and a ventral row of at least 5 simple setae.

**Figure 14.**
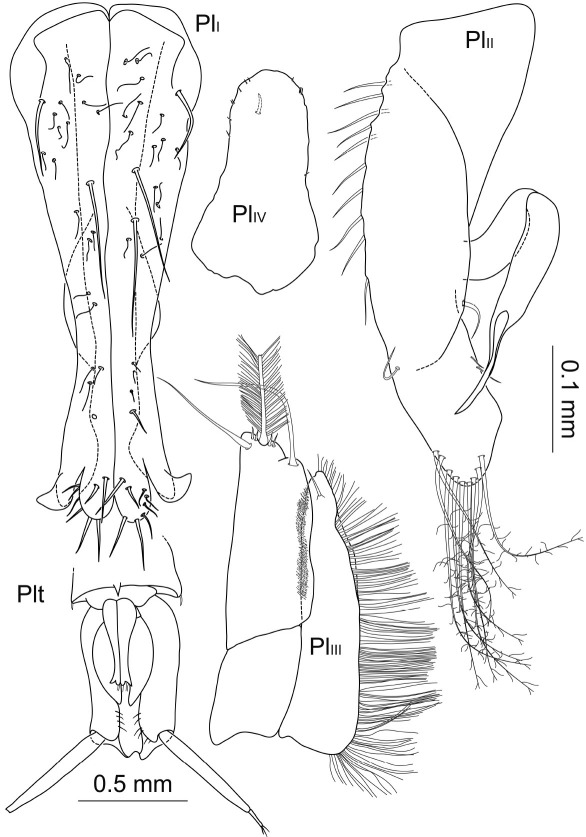
Macrostylis antennamagna sp. n. (Holotype ♂, ZMH (K-42168)) pleopods 1 (ventral view); left pleopods 2–4 (Pl_II_–Pl_IV_, ventral view); setules omitted in 2 of 3 distal plumose setae of pleopod 3 endopod (all pleopods’ scale = 0.1 mm); pleotelson and uropods (ventral view, scale = 0.5 mm, pleopod 1 not planar but obscured projecting into plain of view).

Pleopods 1–4 (Fig. 14) relative length ratios: 1:0.9:0.6:0.4. Operculum of 0.7 times pleotelson length, opening width of distoventral excavation about 0.2 pleotelson width. Pleopod 1 elongate, proximal width about 0.4 times length, width at distolateral lobes 0.3 times length, laterally convex with minimal width about 0.1 times length, setation pattern mostly not symmetrical, distolateral lobes not projecting as wide distally as medial lobes, bent laterally and ventrally, medial lobes rounded, with 7 simple setae of different lengths and strengths arranged symmetrically. Pleopod 2 elongate, proximal width 0.3 times length, maximal width (including stylet) 0.4 times length, stylet almost 0.5 times total length of protopod, laterally with row of at least 11 pappose setae, and 9 long distal pappose setae, medially some simple setae. Pleopod 3 protopod of rhomboid shape, 0.4 times total length, endopod not considerably narrowing, with 3 plumose setae and 2 pairs of 2 short setae distally, exopod 0.9 times total endopod length, distally narrowing, with 1 seta subterminally, row of numerous long simple setae surrounding the exopod laterally incl. distal apex and getting shorter medially. Pleopod 4 protopod and exopod broken off and missing; with several very small setae on apex margin and 1 pronounced seta subterminally.

Uropod (Figs 10, 14) of two articles, 0.8 times pleotelson length, protopod 7.9 times longer than wide, endopod small and slender, less than 0.2 times the length of protopod.

###### Description paratype female.

**Figure 15.**
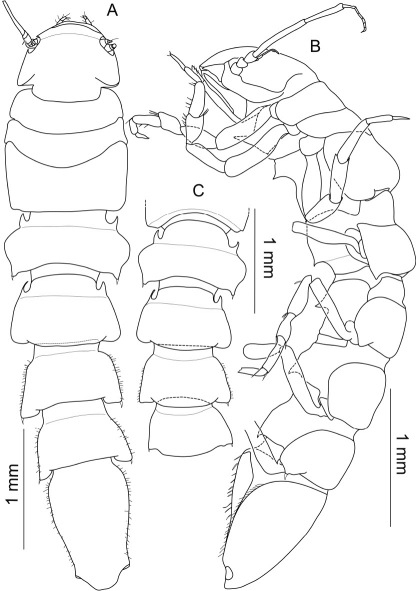
Macrostylis antennamagna sp. n. (Paratype ♀, ZMH (K-42169)) habitus: **A** dorsal view, flattened **B** lateral view **C** dorsal view of pereonites 4–7, bent.

**Figure 16.**
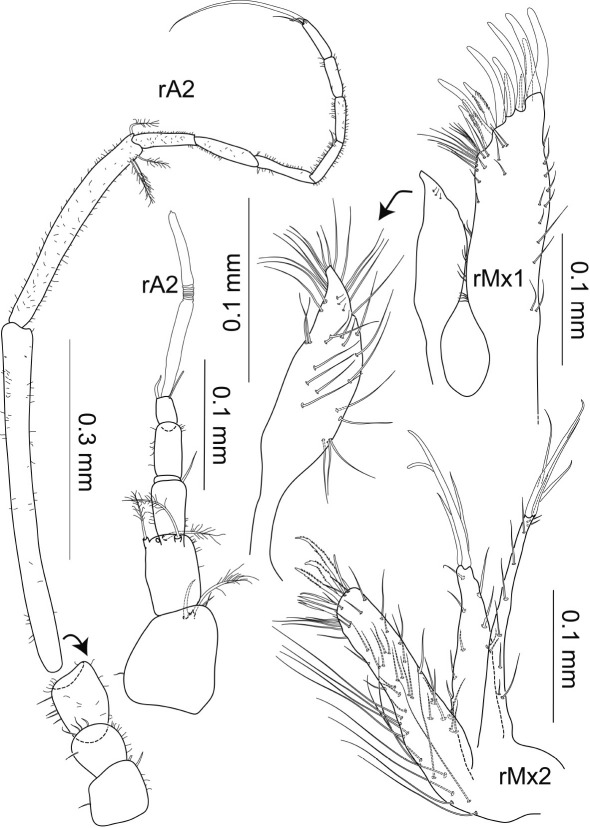
Macrostylis antennamagna sp. n. (Paratype ♀, ZMH (K-42169)) antenna 1 broken in 2 pieces; antenna 2, maxilla 1 (dorsal view); maxilla 2 (dorsal view).

Body (Fig. 15) elongate, 4.5 times longer than wide; maximal body width in pereonite 4, 1.6 times wider than pleotelson; pereonites 2–4 about the same width, 1.1 times pereonite 1 width, small ventral spine posteriorly on pereonite 3. Cephalothorax (Fig. 16) almost semicircular with maximal width at posterolateral corners, length 0.8 times width; slightly longer than 0.1 times total body length; transverse ridge on frons not very prominent, of curved shape. Anterior pereonal division (Fig. 16) 0.2 times total body length; 1.1 times longer than wide. Posterior pereonites (Fig. 16) relative medial segment lengths: 1:1.4:1.1:1.2; relative segment widths of pereonites 4–7: 1:0.9:0.8:0.8, pereonite 7 0.8 the width of pereonite 1. Pleotelson (Fig. 16) 0.2 times body length; about 1.7 times longer than wide; caudal apex slightly concave; breathing cavity maximal opening width 0.7 times pleotelson width, longitudinal excavation 0.2 times maximal pleotelson width.

Antenna 1 (Fig. 16) of 5 articles, 3.4 times longer than article 1 width; articles 1 and 5 as long as wide, fifth article 0.1 times total length, articles 2–4 2 times longer than wide, gradually decreasing in size; 1 aesthetasc on article 5, 0.6 times the length of antenna 1.

Antenna 2 (Fig. 16) relatively slender compared to male antenna 2; articles 1–3 as long as wide; article 4 half as wide as articles 1–3; article 5 0.6 times article 4 length and of same width.

Maxilla 1 (Fig. 16) inner endite shorter and more slender than outer endite, terminally narrowing, around distal apex, dorsally and along lateral margin with numerous long and some very small simple setae; outer endite 1.4 times longer than inner one, narrowing distolaterally, with at least 11 simple setae on lateral margin, with 4 combs of 2–4 simple setae on medial margin, numerous simple setae of different lengths distomedially and 12 robust distal setae, some two-sided denticulate on distal margin.

Maxilla 2 (Fig. 16) inner endite broadest, outer endite of intermediate width, medial endite longest, outer endite 0.8 times middle-endite length, inner endite 0.9 times middle-endite length; margin of inner endite with 12 long simple basomedial setae of more than half the length of endite, numerous small simple setae ventrally and laterally, distal margin with 8 robust setae: 5 simple and 3 denticulate; medial endite with 1 lateral row of simple setae, distally with 4 long simple setae; outer endite with small simple setae along both margins, distally with 4 stiff simple setae of different lengths, the longest of which is of more than half the length of the outer endite. Measurements of article lengths ratios and length-width ratios of articles are listed in Table 3.

**Table 3.** Macrostylis antennamagna sp. n. paratype; preparatory female ZMH (K-42169); relative length ratios of articles of pereopods 1–7 (PI–VII) (basis to dactylus, excluding setae); length-width ratios (L/W) of pereopodal articles; basis damaged in pereopod 2; pereopod 7 broken off and missing.

	Article length ratios	L/W basis	L/W ischium	L/W merus	L/W carpus	L/W propodus	L/W dactylus
P_I_	1 : 0.5 : 0.4 : 0.4 : 0.3 : 0.3	4.1	2.9	2	2.7	3.8	4.7
P_II_	???	???	3.2	2	2.7	2.6	4.6
P_III_	1 : 0.6 : 0.6 : 0.5 : 0.3 : 0.2	3.9	2.2	2.4	3.1	4.3	7.5
P_IV_	1 : 0.4 : 0.4 : 0.3 : 0.2 : 0.1	4.8	2.6	2.1	2.8	2.5	2
P_V_	1 : 0.5 : 0.4 : 0.5 : 0.4 : 0.2	6.2	3.1	2.4	4.8	6.3	5
P_VI_	1 : 0.6 : 0.5 : 0.5 : 0.4 : 0.2	5.2	2.8	2.8	7.7	8.3	3.3

**Figure 17.**
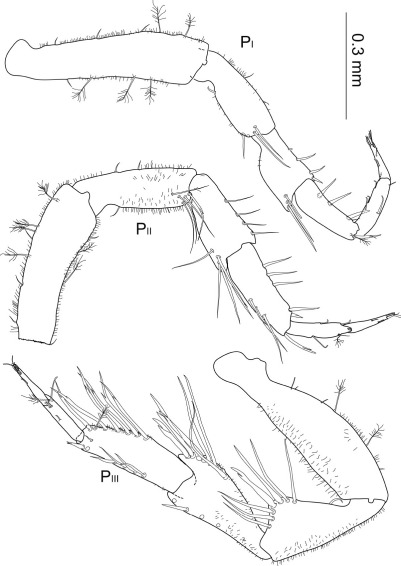
Macrostylis antennamagna sp. n. (Paratype ♀, ZMH (K-42169)) pereopods 1–3 (P_I_–P_III_), pereopod 2 basis broken off.

Anterior pereopods (Fig. 17) increasing in length, relative length ratios, omitting basis: 1:1.2:1.3, with basis longest article. Shapes and setation as in holotype. Pereopod 2 basis damaged. Except: pereopod 3 relative length ratios and setation different from holotype: basis relatively elongated; ischium only 2.2 times longer than wide, dorsal expansion less prominent than in holotype; propodus 0.5 times the length of carpus; dactylus long and slender, 0.9 times carpus length; lengths and robustness of setae as well as the number of setae per row increased in merus and carpus.

**Figure 18.**
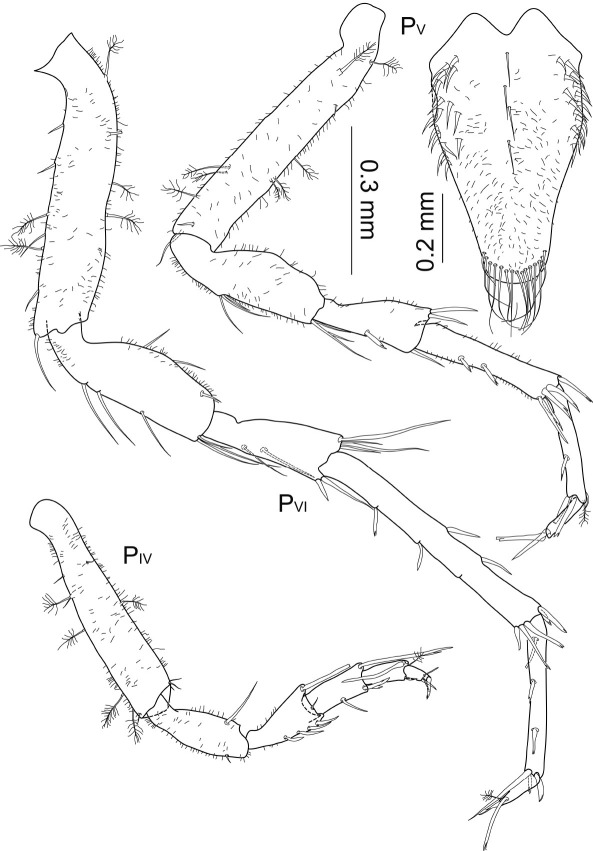
Macrostylis antennamagna sp. n. (Paratype ♀, ZMH (K-42169)) pereopods 4–6 (P_IV_–P_VI_), pereopod 7 missing (scale = 0.3 mm); operculum (ventral view, scale = 0.2 mm).

Posterior pereopods (Fig. 18). Pereopod 4 shortest, length ratios: 1:1.6:2.0; pereopod 7 missing. Pereopod 4 0.7 times pereopod 1 length; dactylus 0.6 times propodus length. Pereopod 5 1.1 times longer than pereopod 1; all articles elongated compared to holotype, especially carpus and propodus. Pereopod 6 1.4 times longer than pereopod 1; with more setae in carpus and propodus.

Operculum (Figs 9, 18) length/width ratio 1.6, densely covered with small setules, with 4 setae alternating along medial line of fusion, numerous setae ventrolaterally along proximal margin, distally with 15 pappose setae. Pleopod 3 and 4 as in male (Fig. 14). Uropods broken off and missing in analysed specimens.

###### Sexual dimorphism.

The male specimen is more slender than the female, its pleotelson is less narrowing posteriorly. The antenna 1 is relatively smaller but wider than in female and with more aesthetascs. The antenna 2 is little larger in the male, almost 0.4 times the length of the total body, 0.3 in female. Pereopods are dimorphic by means of article length ratios while pereopod length in relation to body length and setation are identical in both sexes.

###### Etymology.

“Antennamagna” is derived from the Latin word for “big antenna” as the second antennae are conspicuously large in both sexes.

###### Remarks.

Macrostylis antennamagna sp. n. can be delimited from all other species by the large antenna 2, the three-lobed pars incisiva, the roundish appearance of the cephalothorax without a transverse ridge on frons and the shape of male pleopod 1. Macrostylis antennamagna sp. n. is most similar to Macrostylis urceolata Mezhov, 1989 which is the only known species of this genus with comparably prominent antenna 2. Both species also share the general appearance in dorsal view and have high similarities in following characters: mandibles, male pleopod 1 and ventral spines. Macrostylis antennamagna sp. n. can be distinguished from Macrostylis urceolata by the transverse ridge on frons lacking, antenna 1 being stouter, lacinia mobilis being smaller, the male pleopod 1 being wider and stouter, the smaller relative length of the pleotelson, and the “bifurcate” caudal tip of the pleotelson. Macrostylis gerdesi (Brandt, 2002), Macrostylis carinifera Mezhov, 1988, Macrostylis grandis Birstein, 1970, Macrostylis hirsuticaudis Menzies, 1962, Macrostylis longiremis (Meinert, 1890), Macrostylis minuscularia Mezhov, 2003, Macrostylis sarsi Brandt, 1992, Macrostylis sensitiva Birstein, 1970, Macrostylis ovata Birstein, 1970, and Macrostylis vinogradovae Mezhov, 1992 have a comparably long antenna 2. However, these species show distinct characters clearly delimitating them from Macrostylis antennamagna sp. n. of which only the most obvious are listed below: antenna 1 of Macrostylis gerdesi has nine articles, Macrostylis carinifera bears a posterior ventral spine on pereonite 4 and a much more stout pleopod 2, Macrostylis grandis and Macrostylis ovata have a much wider habitus in dorsal view, Macrostylis hirsuticaudis has an uniquely shaped pleotelson with an almost straight posterior end, the habitus of Macrostylis longiremis is constantly narrowing towards posteriorly and the cephalothorax bears spines in posterolateral corners, Macrostylis minuscularia has a comparably long but thinner antenna 2 and antenna 1 differs in article length ratios and size, Macrostylis sarsi shows a stouter habitus and more slender antenna 2, the latter is also true for Macrostylis sensitiva and Macrostylis vinogradovae.

Antenna 2 can be found even bigger in Macrostylis spinifera Sars, 1864, Macrostylis polaris Malyutina & Kussakin, 1996 and Macrostylis porrecta Mezhov, 1988. However, in Macrostylis spinifera shape and size ratios of male and female pleopods 1 and 2 differ from Macrostylis antennamagna sp. n. and in the other two species antenna 2 has more slender peducular articles and antenna 1 is bigger compared to Macrostylis antennamagna sp. n.

The slender habitus with almost parallel sides in dorsal view shown by Macrostylis antennamagna sp. n. is common in Macrostylidae. The “bifurcated” caudal end is also present in Macrostylis bifurcatus Menzies, 1962, although much stronger developed there. The pleopods 5 have not been found in both sexes. It is unclear, though, whether these have been broken off during dissection or if they are generally reduced in this species. The analysis of five specimens from two stations has revealed little individual variation. Variations in setal counts are probably allometric (compare Hessler 1970 for Desmosomatidae).

##### Macrostylis obscurus

(Brandt, 1992) comb. n.

Fig. 19

Desmostylis obscurus Brandt, 1992, p. 69–74, Fig. 11–13

###### Additions to original description.

Pereopod 4 relative article length ratios: 1:0.5:0.5:0.4:0.3:0.1, basis 2.3 times longer than wide, with at least 5 broom setae, 1 short simple seta distodorsally; ischium 1.8 times longer than wide, with 1 short simple seta distodorsally; merus almost 1.8 times longer than wide, distodorsally with 1 stout bifurcate seta, distoventrally 2 acute simple setae, 1 reaching beyond propodal articulation; carpus 1.6 times longer than wide, distodorsally with 1 bifurcate seta, 1 broom seta distoventrally next to 1 long simple seta exceeding dactylus in length; propodus twice as long as wide, dorsally expanding into a distal lobe, with 1 long bifurcate seta distoventrally, exceeding dactylar claw, and 1 short and simple seta ventrally; dactylus small, twice as long as wide, about half propodus length and width, with 1 terminal stout and acute claw, 1.5 times article length, and 1 subterminal bifurcate claw, shorter than article, 2 setae terminally and 1 thin and long simple seta subterminally, twice as long as article.

Pereopod 5 propodus 3.3 times longer than wide, with 2 long simple setae distoventrally, thinner seta more than twice as long as dactylus, the stouter one almost reaching the tip of dactylus, 1 simple seta ventrally; dactylus 5 times longer than wide, half propodus length, with 3 simple, acute claws, terminal claw longer than dactylus, subterminal claw stout, robust and shorter than dactylus, second subterminal claw slender and more than twice as long as dactylus.

Pereopod 6 propodus expanding into a distal lobe, with 1 long bifurcate seta, reaching beyond tip of dactylus, with 1 very long and simple seta and 1 broom seta; dactylus 4.3 times longer than wide, with 1 terminal long and acute claw, 1.5 times dactylus length, and 1 subterminal claw, longer than article, with 2 thin and unequally long simple setae subterminally.

**Figure 19.**
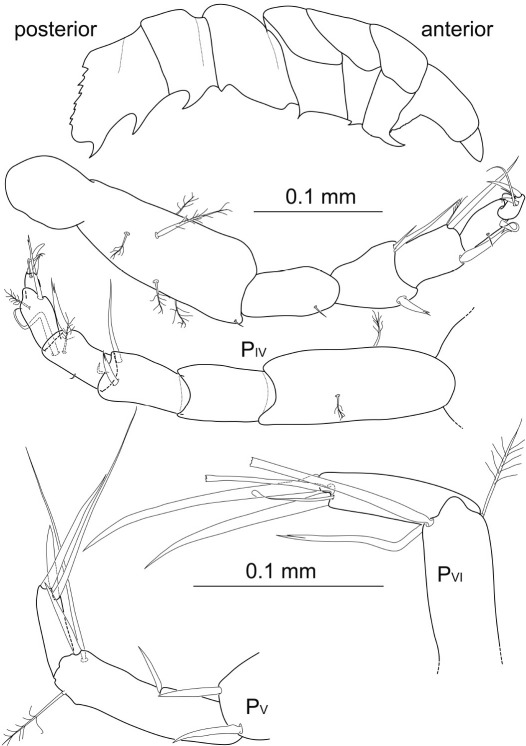
Top: Macrostylis gerdesi (Brandt, 2002) comb. n., holotype female (ZMH (K-39915)); lateral view on left side; specimen damaged: pereonite 7 broken off, pereopods, antennae 1–2 and mouthparts dissected; ventral spines on pereonites 1–3 and 5–7 (for measurements see original description); below: Macrostylis obscurus (Brandt, 1992) comb. n. holotype female manca, (BNHM 1990:39); left and right pereopods 4; dactyli of pereopods 5 and 6.

##### Macrostylis gerdesi

(Brandt, 2002) comb. n.

Fig. 19

Desmostylis gerdesi Brandt, 2002, p. 616–626, Fig. 1–4.

###### Additions to original description.

Ventral spine in pereonite 1, directed onteriorly; ventral spines in pereonites 2–3 and 5–7, directed posteriorly. Pereonites 4–6 slightly constricted towards anterior margin.

###### Remarks.

Re-examination revealed spines on all sternites not illustrated before, except from pereonite 4. In the latter a ventral constriction is present close to the posterior margin. In Macrostylis gerdesi comb. n. long sensory terminal setae on dactyli and additionally long sensory setae on propodi of posterior pereopods are considered to be apomorphies for Desmostylis. The value of these characters to discriminate between genera is discussed above. However, a dactylus is present in pereopod 4 and in contrast to the Macrostylis obscurus comb. n. holotype, pereopod 3 ischium is triangularly expanded in Macrostylis gerdesi comb. n. As a consequence of the character discussion listed above this species is transferred the genus Macrostylis.

## Discussion

Pereopodal measures are variable within a species. Not only can differences be found between the sexes (see below) but also within a sex. Certain variability does not necessarily occur in all pereopods at the same time and to the same extend. In Macrostylis uniformis sp. n., for example, we have found the pereopod 3 to be almost similar with regard to length-width ratios in an ovigerous and a non-ovigerous female specimen. Here, only the number of setae per row was slightly different. Contrastingly, pereopod 5 shows rather strong variation. While the pereopod 5 in general and most of its articles were longer in the ovigerous female, ischium and merus had the same length compared to the preparatory female. Besides propodus and dactylus, all articles had the same width or were narrower in the ovigerous female compared to the preparatory one. These measures could be interpreted as allometry but may also be variable within a stage.

In Macrostylis antennamagna sp. n. we observed differences in posterolateral setation of cephalon and pereomeres with regard to robustness, length and substructures. These differences occurred between male and female as well as between left and right side of the same pereomere of one specimen. This indicates that such setal features are intraspecifically variable. To clarify if this is general variability or sexual dimorphism, higher numbers of specimens need to be analysed.

Sexual dimorphisms have been reported for a wide range of taxa. Not much is known about dimorphism in Macrostylidae. Here, species have been described based on one sex only. Sexual dimorphism is probably the reason why correct allocation of a complementary male or female is often impossible based solely on morphological characters. This is the case for example in Macrostylis uniformis sp. n. Knowledge about general patterns of dimorphism in closely related species could provide an aid for allocation in new species. However, we found distinct differences between males and females in macrostylids. A general pattern of sexual dimorphism can be found in the copulatory organs and a few additional characters: the male antenna 1 bears more aesthetascs and is sometimes broader than in the female (Hansen 1916, Menzies and George 1972). Furthermore, in the antenna 2 articles are sometimes broader in males than in females (Mezhov 2003a, b).

To date no cases of more extreme sexual dimorphism, as for example reported for the paramunnid genus Abyssaranea Wilson & Hessler, 1974, have been described in Macrostylidae. It is possible that no example has been discovered so far but it also may be that in strongly dimorphic species males and females have been defined as distinct species. Wilson and Hessler (1974) mentioned the possible value of the dimorphic degree as being a significant character on generic level. A comprehensive comparison of gender differences has been reported for Macrostylis dellacrocei Aydogan, Wägele & Park, 2000. In the original description a juvenile male was compared to the preparatory female holotype. Differences found exceed the above mentioned general pattern for Macrostylidae by far. The differences that occurred between sexes (i.e. lack of sternal projection in pereonite 1, the relatively smaller pereonite 7, the smaller aesthetasc and terminal article in the antenna 1, the reduced sizes of pars molaris and spine row in the mandible, the reduced sizes in right and left lacinia mobilis, reduced number of setae on pereopod 7), though, are most likely allometric characters at an early ontogenetic stage in the juvenile male specimen. Therefore, these characters cannot be used to infer sexual dimorphism in this species or to gain insights into general patterns of sexual dimorphism in Macrostylidae.

On this background we assume sexual dimorphism likely to be common in Macrostylidae and to vary from species to species. Both sexes have so far only been described in species with low degree of sexual dimorphism, where allocation was straight forward. It is likely that in some cases only one gender per species has been collected, particularly where sample size is low. It may also be likely that strong sexual dimorphism leads to allocation of male and female specimens into separate species. This could e.g. be the case in Macrostylis uniformis sp. n. To allocate specimens safely it is necessary to know characters that are less affected by sexual dimorphism. For the identification of such characters, analyses of higher numbers of specimens from one sampling site are needed. Patterns could be not only generalized to allocate species where lower numbers of specimens are available but probably also be used to infer subtaxa (i.e. genera) within Macrostylidae.

## Supplementary Material

XML Treatment for Desmosomidae
